# Supplementary material for: Analysis of off-tumour toxicities of T-cell-engaging bispecific antibodies via donor-matched intestinal organoids and tumouroids
Source: Nat Biomed Eng. 2023 Dec 19;8(4):345–60. doi: 10.1038/s41551-023-01156-5 (PMC11087266; doi:10.1038/s41551-023-01156-5)
Supplement: Supplementary file 1 — Supplementary figures and tables. [file 41551_2023_1156_MOESM1_ESM.pdf]

nature biomedical engineering

Article

https://doi.org/10.1038/s41551-023-01156-5

Analysis of off-tumour toxicities of T-cell-engaging bispecific antibodies via donor-matched intestinal organoids and tumouroids

In the format provided by the authors and unedited

Supplementary figures

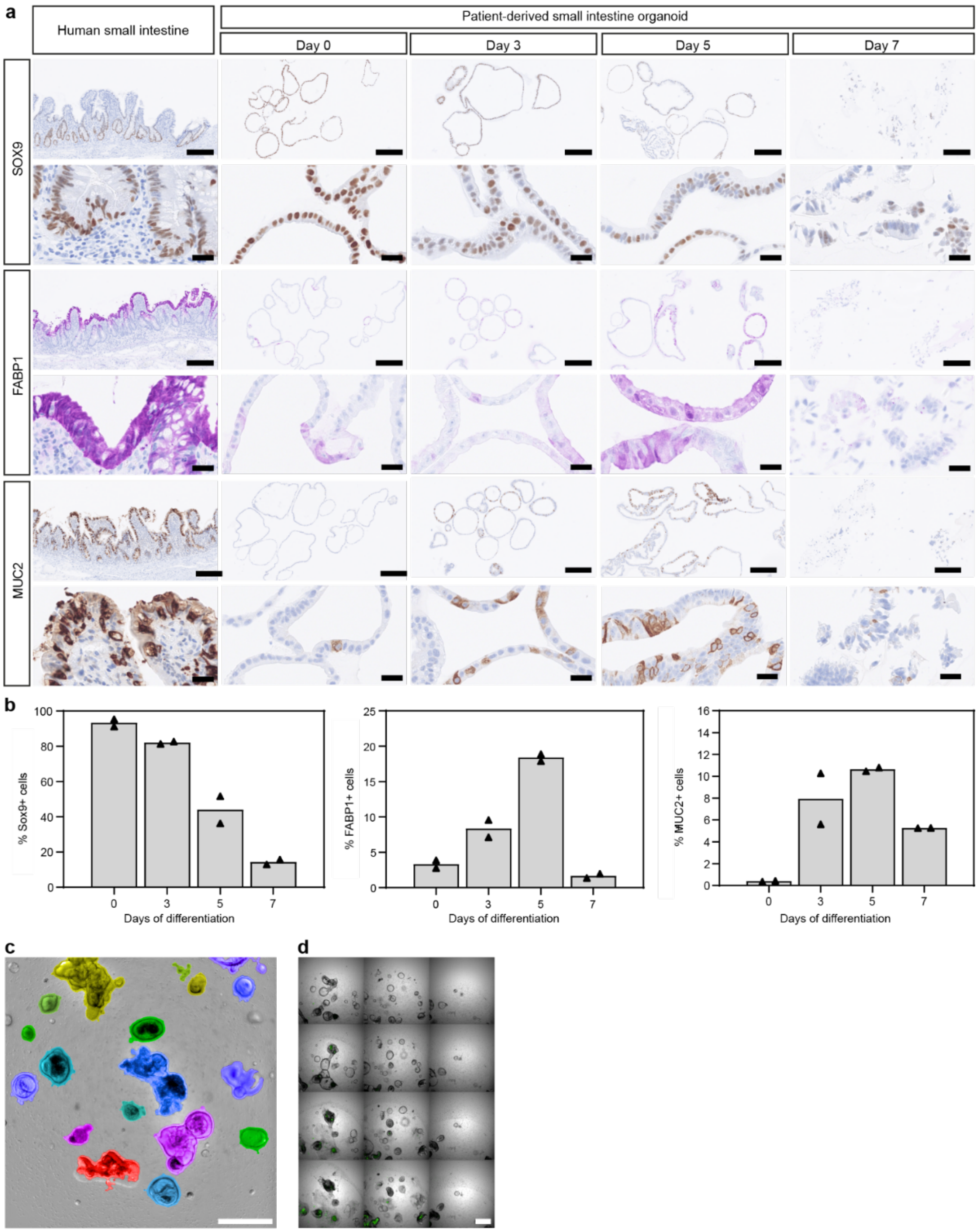

Supplementary. Fig. 1 I Immunohistochemical characterization of intestinal organoids over time and comparison to human native tissue and low-resolution image-based killing assay. a, Expression of SOX9, FABP1 and MUC2 in human small intestine tissue and patient-derived organoids. Organoids were grown for the according fixation time point (Day 0, 3, 5, 7; n=2 per time point). Day 0 relates to one week of cultivation with organoid growth medium and represents the undifferentiated, crypt-like state intestinal epithelium. For day 3-7, the organoids were switched to differentiation medium and cultivated further until the indicated time. Intestinal stem cells were detected by SOX9+ DAB staining. Enterocytes were identified by purple FABP1+ staining, goblet cells by brown MUC2+ staining. Scale: 250 μm. Magnified insert scale: 25 μm. **b**, Quantitative image analysis of the makers in **a** was performed on all organoids per slide. Graphs depict mean positive cells per total cells ∓ SD. n=2 per marker. **c**, Representative image of organoid segmentation for subsequent caspase-3/7 quantification within. Scale: 500 μm. **d**, Representative single tiles of merged bright-field and caspase-3/7 IF (green) images of the untreated co-culture control over a time course of 72 h at 5x magnification. Scale per tile: 500 μm. The experiments represented in **c-d** in this figure were replicated a minimum of five times, yielding similar results.

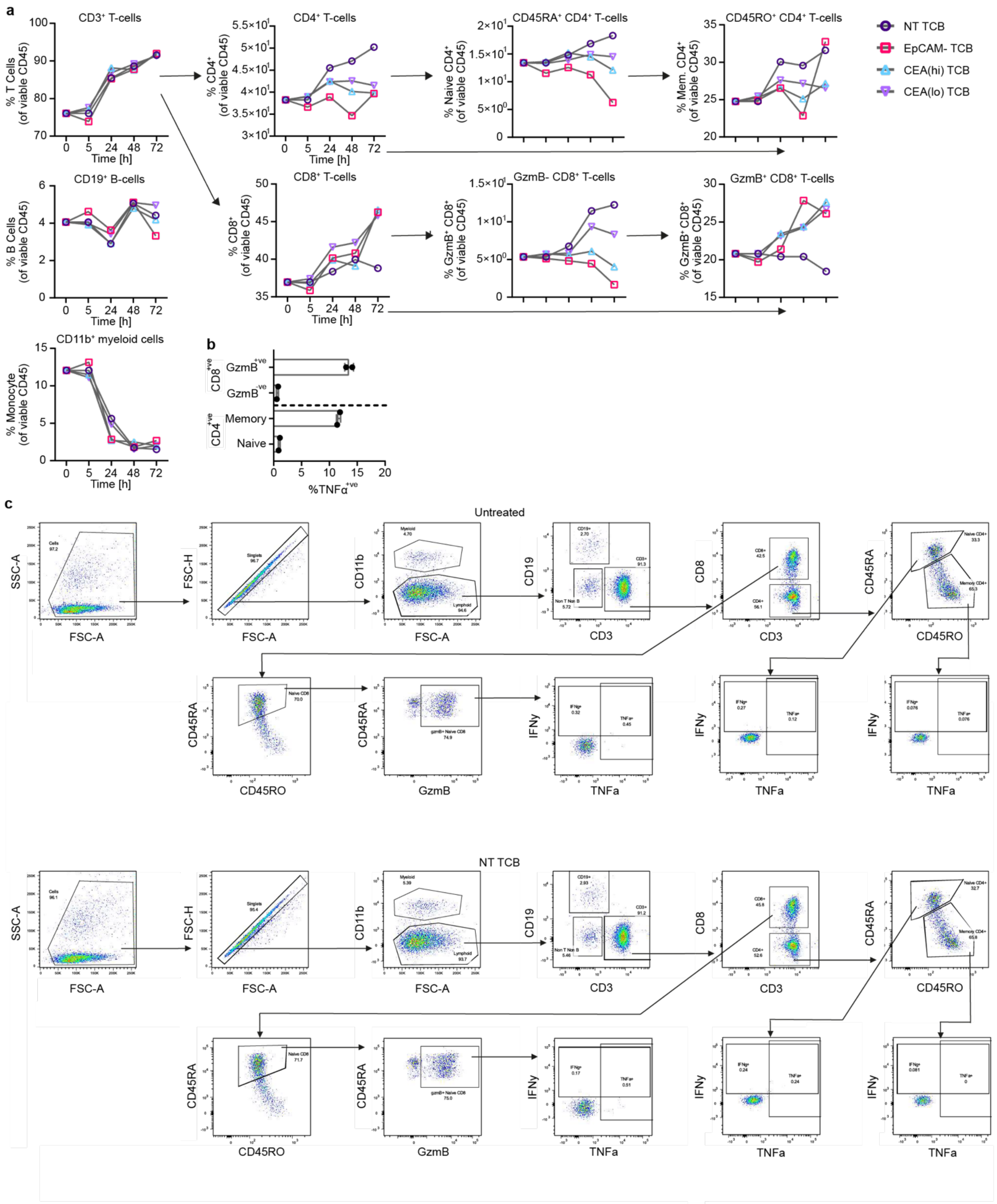

Supplementary Fig. 2 | Changes of immune populations upon TCB stimulation. a, Assessment of immune population fate upon across all TCBs (10 μg/mL) over time. **b,** Percentage of intracellular TNFα expression in GzmB+ and GzmB- CD8+ T cells and memory (CD45RO+) and naïve (CD45RA+) CD4 T cells treated 24 h post CEA(hi) TCB treatment. Data are presented as mean values ∓ SD (n=2)**. c**, Comparison of untreated control vs. NT TCB control at 24 h post treatment. This panel also exemplifies the main gating strategy used to generate the flow cytometry results presented in the manuscript.

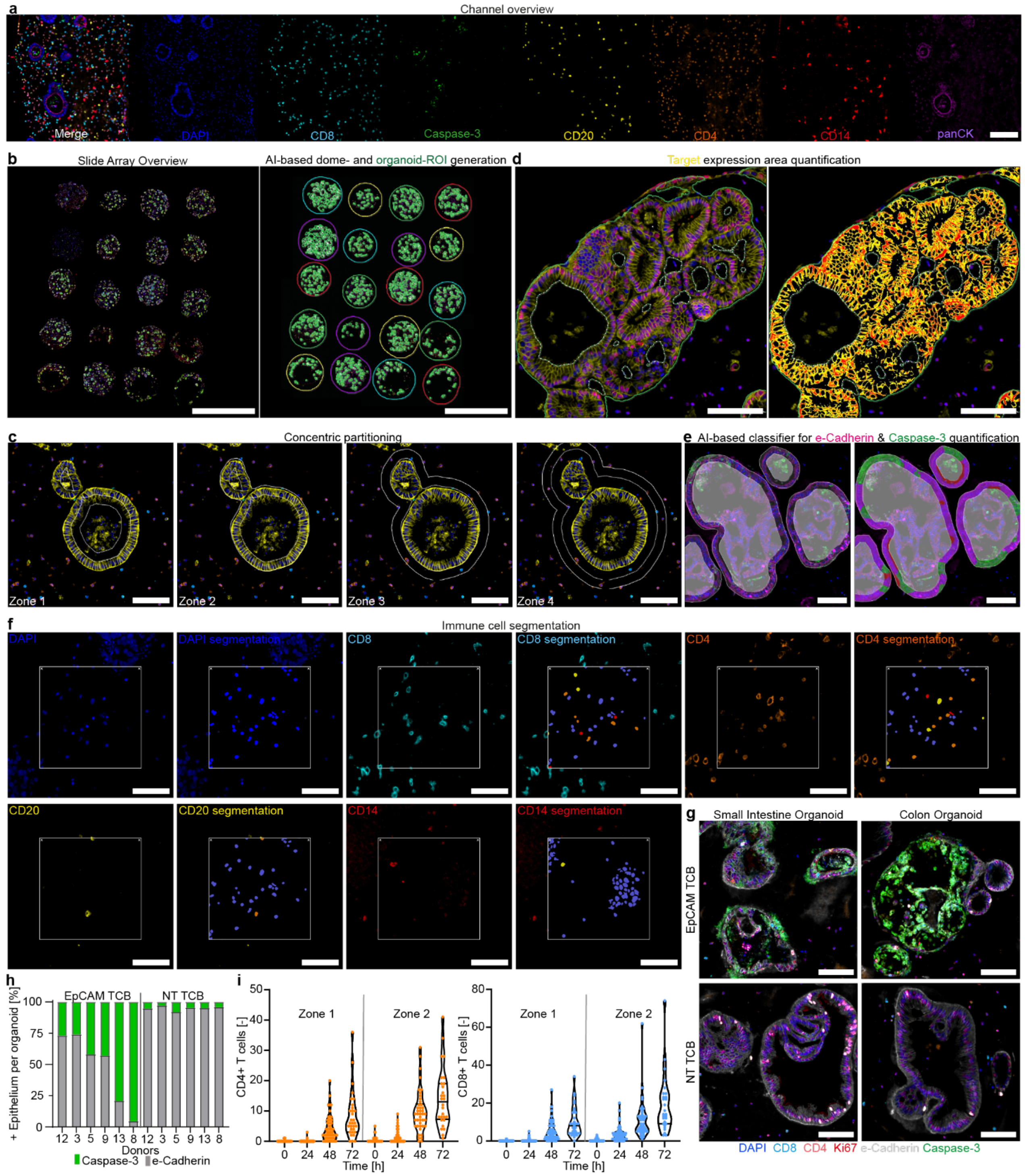

Supplementary Fig. 3 | Organoid-PBMC co-culture mIF image-analysis. a, Representative merged image plus individual channels of the 7-plex mIF images. PanCK+ organoids are surrounded by CD4+ (orange), CD8+ (turquoise), CD14+ (red) and CD20+ (yellow) immune cells. Caspase-3 (green) captures apoptosis. Nuclei are stained with DAPI (blue). Scale: 100 μm (applies to each individual channel). **b,** Annotation of cross-sectioned array of domes allows automated generation of annotations of single organoids (>7500 μm^2) within using a pre-trained AI-classifier. **c,** Initial ROI of objects (Zone 2) enabled generation of further outward and inward regions around ROI of the organoid. Scale: 1 mm. **d,** Representative IF of EpCAM+ area quantification. Red indicates high EpCAM+ optical density, yellow represents lower levels. Dashed lines around empty space (lumen) highlights excluded area within object. Scale: 100 μm. **e,** Segmentation of E-Cadherin+ and caspase-3+ positive area in zone 2 of the organoid based on a pre-trained AI-classifier. Scale: 100 μm. **f,** Segmentation of individual immune subsets per channel. Red, orange and yellow reflect different thresholds for segmentation positivity. Blue indicates segmented cells. Scale: 100 μm. **g,** Extent of damage highlighted by representative SI and colon organoids treated with EpCAM and NT TCB. Scale: 100 μm. **h,** Sum of E-Cadherin+ (grey) and caspase-3+ (green)

epithelium of organoids detected in zone 2 (epithelium only) at 48 h time point (n≥3). **i,** T cell infiltration number per individual organoid (n≥30) of $CD4^+$ and $CD8^+$ T lymphocytes in zone 1 and 2 across time treated with EpCAM TCB (10 μg/mL). All of the displayed experiments in this figure were replicated a minimum of five times, yielding similar results.

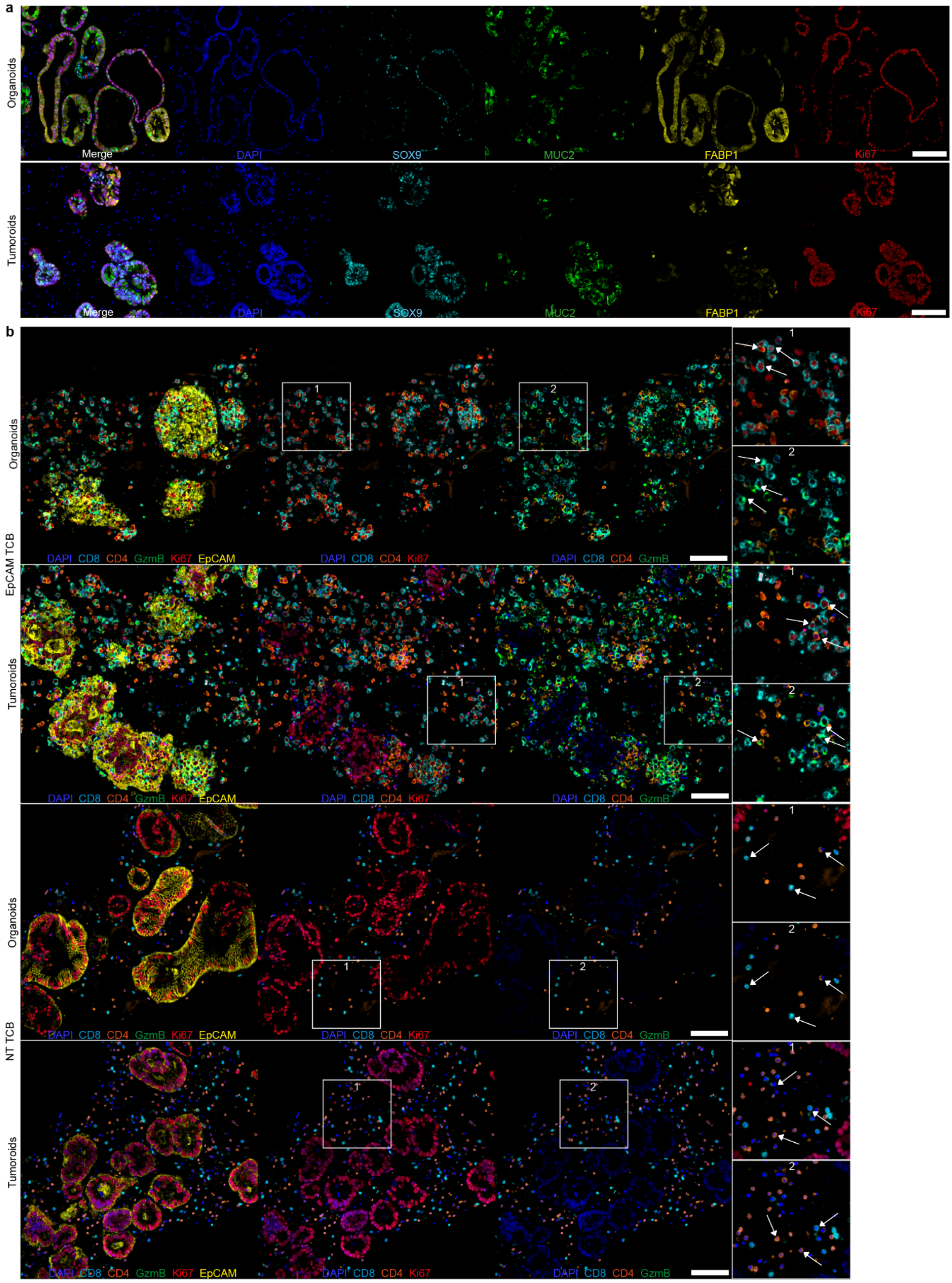

Supplementary Fig. 4 I Characterization of organoids and tumoroids, GzmB and Ki67 induction in T cells regardless of vicinity to the target. a, Expression profile of the major functional cell types in 3-day-differentiated donor-matched colon organoids and tumoroids by 5-plex mIF imaging at 20x magnification. FABP1+ enterocytes (yellow) and MUC2+ goblet cells (green) in co-existence with crypt-associated cells such as SOX9+ stem cells (turquoise) in their Ki67+ proliferative state (red), merged with DAPI+ nuclei (blue). Scale: 100 µm. **b**, 6-plex mIF images at 15x magnification of donor-matched colon organoids and tumoroids co-cultured with PBMCs, 72 h post administration of NT and EpCAM TCB. CD8+ T cells (turquoise) and CD4+ T cells swarm the EpCAM+ organoids and tumoroids (yellow), respectively. Within EpCAM (TCB) condition, both T-lymphocytes subsets are highly proliferative (Ki67+, red) and exhibit high levels of GzmB (green). Scale: 100 µm.). The displayed experiment in this figure was replicated once, yielding similar results.

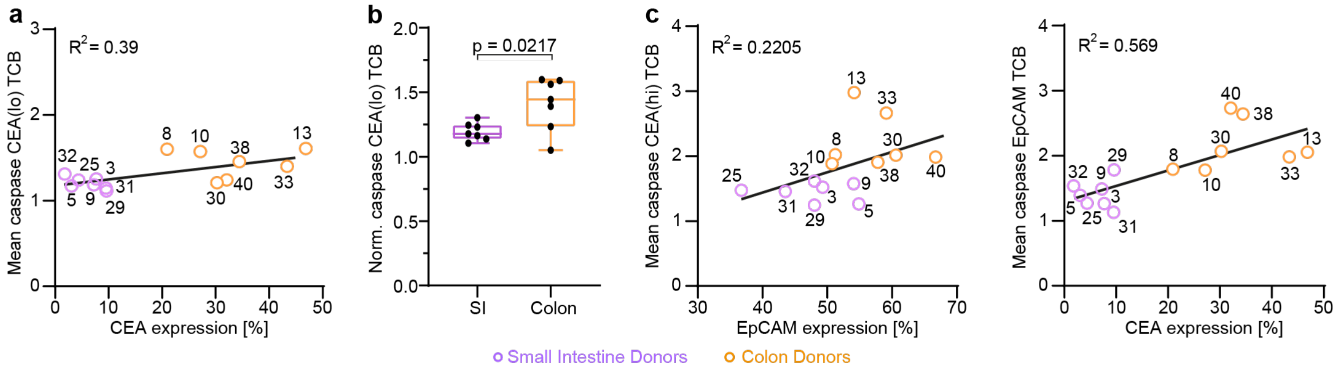

Supplementary Fig. 5 I Correlation between target expression and effector outcome. a, Correlation plots between target expression of CEA and EpCAM and normalized caspase-3/7 signal of the respective TCB. R-squared is provided per plot. **b**, Data as boxplot, whiskers showing all points (min to max) of the mean of normalized caspase-3/7 across all donors distinguished between the respective intestinal regions for each organoid line. Unpaired t-test (two-tailed) was performed and was defined as *p<0.05 (n=7). **c**, Correlation plot between CEA TCB-induced apoptosis and EpCAM target expression as well as EpCAM TCB-mediated killing and CEA target expression, 48 h post administration. R-squared is provided per plot.

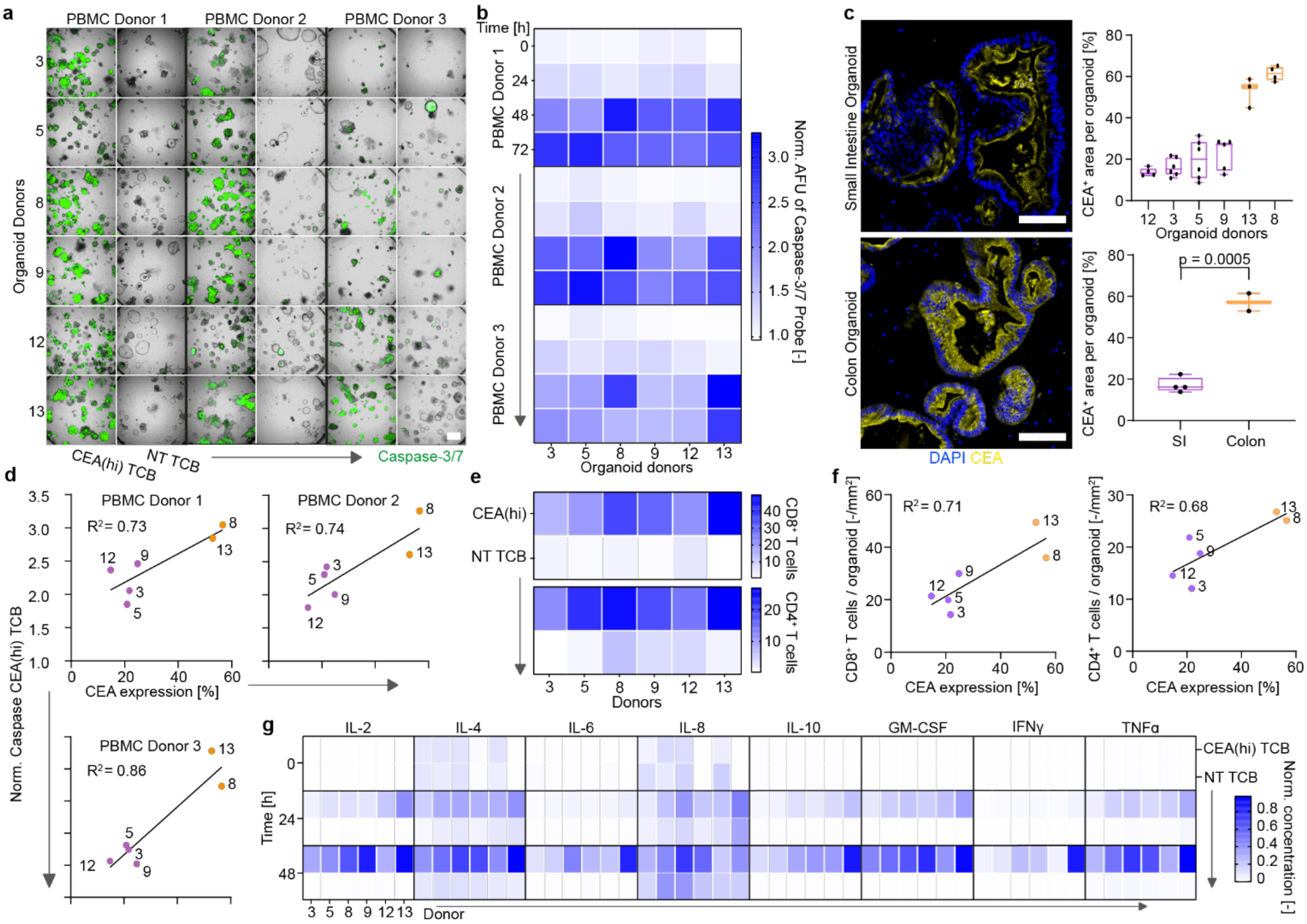

Supplementary Fig. 6 I Effect of PBMC donor on TCB-triggered organoid damage. a, Representative single tiles of merged bright-field and caspase-3/7 IF (green) images of the six organoid donors co-cultured with three different PBMC donors treated CEA(hi) and NT TCB (0.5 μg/mL) over a time course of 72 h at 5x magnification. Scale per tile: 500 μm. **b,** Heat map of quantified caspase-3/7 fluorescence signal in individual organoids for each TCB treatment across time, per well and condition (n=3). Mean fluorescence signal for each TCB-condition normalized to the mean AFU detected in the NT TCB control at each time point. The 6 different patient-derived organoid lines are displayed on the x-axis, the PBMC donors on the y-axis. **c,** Representative IF image of CEA expression of a SI and colon organoid. Imaged at 20x, scale: 100 µm. CEA target expression per organoid donor as well as per anatomical origin based on CEA area quantification per individual objects. Data as boxplots, whiskers showing all points (min to max; n_{Don12}=4, n_{Don3}=6, n_{Don5}=6, n_{Don9}=5, n_{Don13}=3, n_{Don8}=4; $n_{SIDonors}$=4, $n_{ColDonors}$=2). Unpaired t-test (two-tailed) was performed and was defined as ***p<0.001. **d,** Correlation plot between CEA TCB-induced apoptosis (Norm. caspase-3/7 AFU) and CEA target expression per PBMC donor, 48 h post administration. R-squared is provided per plot. **e,** CD8+ T cell infiltration and CD4+ T cell infiltration (PBMC Donor 3) per organoid, plotted as mean (n=3; normalized for area of objects). **f,** Correlation plot between CD8+ and CD4+ T cells (PBMC Donor 3) versus EpCAM expression across all organoid donors at 48 h post, normalized to organoid area. R-squared is provided per plot. **g,** Heat map of multiplex cytokine analysis performed on supernatants from treated wells across all time points and TCBs administered. Normalized per cytokine, mean per condition plotted (n=3). The displayed experiment in this figure was replicated once, yielding similar results.

Supplementary tables

Supplementary table 1 I Donor information for Intestinal resection.

Donor number	Anatomical origin	Sex	Age	Biopsy origin
3	Jejenum	Female	83	HTCR
4	Colon	Female	30–39	HTCR
5	Duodenum	Female	40–49	HTCR
8	Colon ascendens	Female	40–49	HTCR
9	Duodenum	Male	40–49	HTCR
10	Sigmoid Colon	Male	50–59	HTCR
12	Tumour sigmoid colon and normal sigmoid colon	Male	50.59	HTCR
13	Colon ascendens	Male	60–69	HTCR
25	Duodenum	Male	80–89	HTCR
29	Jejenum	Female	76	HTCR
30	Rectum	Female	59	HTCR
31	Jejenum	Male	78	HTCR
32	Duodenum / Jejenum	Male	77	HTCR
33	Rectum	Male	49	HTCR
38	Sigmoid Colon	Female	81	HTCR
P464	Tumour colon and normal colon	Male	60	University Hospital Basel
P471	Tumour rectum and normal rectum	Female	70	University Hospital Basel
P634	Tumour colon and normal colon	Male	76	University Hospital Basel
P645	Tumour colon and normal colon	Male	51	University Hospital Basel

Supplementary table 2 | Flow-cytometry antibodies.

Fluorophore	Antigen	Vendor	Clone	Catalogue
BUV395	IFNγ	BD Biosciences	B27	563563
BUV737	CD69	BD Biosciences	FN50	612817
BUV805	CD8	BD Biosciences	SK1	612889
BV421	Ki-67	Biolegend	Ki-67	350506
BV421	CD103	Biolegend	Ber-ACT8	350214
BV510	CD45	Biolegend	2D1	368526
BV605	CD19	Biolegend	HIB19	302244
BV650	HLA-DR	Biolegend	L243	307650
BV711	CD11b	Biolegend	ICRF44	301344
BV786	CD45RA	Biolegend	HI100	304140
FITC	CD45RO	Biolegend	UCHL1	304204
PE	TNFα	Biolegend	MAb 11	502909
PE-Dazzle-594	Granzyme B	Biolegend	QA16A02	372215
PE-Cy7	41BB	Biolegend	4B4-1	309818
APC	IL-2	Biolegend	MQ1-17H12	500310
Alexa Fluor 700	CD3	Biolegend	HIT3a	300324
APC-H7	Efluor780 Fixable L/D	Thermo	N/A	65-0865-14

Supplementary table 3 I IHC and mIF antibodies.

Application	Antigen	Vendor	Reference	Dilution	Incubation [min]
mIF	Recombinant SOX9	Abcam	Ab1859666	1:800	32
mIF	FABP1	Life Technologies	PAS28945	1:100	32
mIF	MUC2	Life Technologies	MAS512345	1:100	32
mIF	Ki67	Invitrogen	14-569882	1:500	32
mIF	CD3	Ventana	790-4341	prediluted	40
mIF	CD4	Ventana	790-4423	prediluted	40
mIF	CD8	Ventana	790-4460	prediluted	40
mIF	CD14	Abcam	ab181470	1:200	60
mIF	CD20	DAKO	M0755	1:200	40
IHC	Recombinant CD103	Abcam	Ab227697	1:75	52
mIF	Cleaved Caspase-3	Cell Signaling Technology	9661	1:100	40
IHC & mIF	CEA	Abcam	ab207718	1:100	40
IHC & mIF	EpCAM	Ventana	760-4383	prediluted	40
IHC & mIF	E-Cadherin	Ventana	760-4497	prediluted	60
mIF	Pan-Cytokeratin	Biorybt	Orb10399	1:100	60
mIF	Granzyme B	Abcam	Ab4059	1:100	60
IHC	ZO-1	Invitrogen	339100	1:50	60
