# Supplementary material for: Analysis of off-tumour toxicities of T-cell-engaging bispecific antibodies via donor-matched intestinal organoids and tumouroids
Source: Nat Biomed Eng. 2023 Dec 19;8(4):345–60. doi: 10.1038/s41551-023-01156-5 (PMC11087266; doi:10.1038/s41551-023-01156-5)
Supplement: Supplementary file 2 — Reporting Summary [file 41551_2023_1156_MOESM2_ESM.pdf]

nature portfolio

Corresponding author(s): Nikolche Gjorevski

Last updated by author(s): Oct 20, 2023

# Reporting Summary

Nature Portfolio wishes to improve the reproducibility of the work that we publish. This form provides structure for consistency and transparency in reporting. For further information on Nature Portfolio policies, see our Editorial Policies and the Editorial Policy Checklist.

## Statistics

For all statistical analyses, confirm that the following items are present in the figure legend, table legend, main text, or Methods section.

| n/a | Confirmed | |
|---|---|---|
| ☐ | ☒ | The exact sample size (*n*) for each experimental group/condition, given as a discrete number and unit of measurement |
| ☐ | ☒ | A statement on whether measurements were taken from distinct samples or whether the same sample was measured repeatedly |
| ☐ | ☒ | The statistical test(s) used AND whether they are one- or two-sided<br>*Only common tests should be described solely by name; describe more complex techniques in the Methods section.* |
| ☒ | ☐ | A description of all covariates tested |
| ☒ | ☐ | A description of any assumptions or corrections, such as tests of normality and adjustment for multiple comparisons |
| ☐ | ☒ | A full description of the statistical parameters including central tendency (e.g. means) or other basic estimates (e.g. regression coefficient) AND variation (e.g. standard deviation) or associated estimates of uncertainty (e.g. confidence intervals) |
| ☐ | ☒ | For null hypothesis testing, the test statistic (e.g. *F*, *t*, *r*) with confidence intervals, effect sizes, degrees of freedom and *P* value noted<br>*Give P values as exact values whenever suitable.* |
| ☒ | ☐ | For Bayesian analysis, information on the choice of priors and Markov chain Monte Carlo settings |
| ☒ | ☐ | For hierarchical and complex designs, identification of the appropriate level for tests and full reporting of outcomes |
| ☒ | ☐ | Estimates of effect sizes (e.g. Cohen's *d*, Pearson's *r*), indicating how they were calculated |

*Our web collection on statistics for biologists contains articles on many of the points above.*

## Software and code

Policy information about availability of computer code

| | |
|---|---|
| Data collection | No software was used for data collection. |
| Data analysis | Commercial software packages used for data analysis:<br>Mage analysis: HALO AI v3.4.2986.209 (Indica Labs) and Harmony v4.9 (Perkin Elmer)<br>Statistical analysis: GraphPad Prism v8.4.2<br>Flow-cytometry data analysis: FlowJo v10.8.1 |

For manuscripts utilizing custom algorithms or software that are central to the research but not yet described in published literature, software must be made available to editors and reviewers. We strongly encourage code deposition in a community repository (e.g. GitHub). See the Nature Portfolio guidelines for submitting code & software for further information.

## Data

Policy information about availability of data

All manuscripts must include a data availability statement. This statement should provide the following information, where applicable:

- Accession codes, unique identifiers, or web links for publicly available datasets
- A description of any restrictions on data availability
- For clinical datasets or third party data, please ensure that the statement adheres to our policy

The main data supporting the results in this study are available within the paper and its Supplementary Information. Source data for the figures are provided with this paper. The raw and analysed datasets generated during the study are available for research purposes from the corresponding authors on reasonable request.

## Human research participants

Policy information about studies involving human research participants and Sex and Gender in Research.

| | |
|---|---|
| Reporting on sex and gender | The experiments reported in the paper were performed using intestinal resections derived from a nearly equal number of male (11) and female (8) donors (wherein 'male' and 'female' refers to sex) (Supplementary Table 1). Information on the gender of the donors was not available and we are unable to report it. We did not observe sex to have any discernible influence on the main readouts within the study (target expression, TCB-mediated apoptosis/cytokine release/T-cell infiltration), which were strictly governed by variables independent of sex (such as intestinal region, TCB type and concentration). |
| Population characteristics | The information on donor age is included in Supplementary Table 1. Small intestinal donors had been diagnosed with pancreatic cancer or pancreatitis, whereas colon and rectum donors had been diagnosed with colorectal cancer. Genotypic information on driver mutations and treatment history is available on request, but not included in this paper as we deem it not relevant to the main aims and conclusions. |
| Recruitment | We did not devise a prospective recruitment strategy. We included into the study any available surgical specimen of human intestinal tissue during the period over which the study was performed. |
| Ethics oversight | The framework of the HTCR Foundation, which includes written informed consent from all donors, was approved by the ethics commission of the Faculty of Medicine in the LMU (number 025-12) and the Bavarian State Medical Association (number 11142). |

Note that full information on the approval of the study protocol must also be provided in the manuscript.

# Field-specific reporting

Please select the one below that is the best fit for your research. If you are not sure, read the appropriate sections before making your selection.

☒ Life sciences ☐ Behavioural & social sciences ☐ Ecological, evolutionary & environmental sciences

For a reference copy of the document with all sections, see nature.com/documents/nr-reporting-summary-flat.pdf

# Life sciences study design

All studies must disclose on these points even when the disclosure is negative.

| | |
|---|---|
| Sample size | No statistical methods were used to predetermine sample sizes. Sample sizes used were deemed to be representative and sufficient, on the basis of the low variability of the results between technical and biological replicates and on overall reproducibility. |
| Data exclusions | No data were excluded. |
| Replication | All experiments presented in this study were repeated at least three times (most of them more than five times) to ensure the reproducibility of the results. All attempts at replication were successful. |
| Randomization | Randomization was not performed, because we did not identify factors that would co-vary with different treatment conditions within a single experiment. |
| Blinding | Blinding was not performed, as all analyses (flow cytometry, cytokine analysis, image analysis) were performed in bulk, applying the exact same settings for all treatment conditions. |

# Reporting for specific materials, systems and methods

We require information from authors about some types of materials, experimental systems and methods used in many studies. Here, indicate whether each material, system or method listed is relevant to your study. If you are not sure if a list item applies to your research, read the appropriate section before selecting a response.

## Materials & experimental systems

| n/a | Involved in the study |
|---|---|
| ☐ | ☒ Antibodies |
| ☐ | ☒ Eukaryotic cell lines |
| ☒ | ☐ Palaeontology and archaeology |
| ☒ | ☐ Animals and other organisms |
| ☒ | ☐ Clinical data |
| ☒ | ☐ Dual use research of concern |

## Methods

| n/a | Involved in the study |
|---|---|
| ☒ | ☐ ChIP-seq |
| ☐ | ☒ Flow cytometry |
| ☒ | ☐ MRI-based neuroimaging |

# Antibodies

Antibodies used

1. IHC Antibodies: Antigen, Vendor, Clone, Dilution, Reference;
Recombinant SOX9, Abcam, EPR14335-78, 800, Ab1859666; FABP1, Life Technologies, N/A, 100, PA5-28945; MUC2, Life Technologies, 996/1, 100, MA5-12345; Ki67, Invitrogen, SolA15, 500, 14-569882; CD3, Ventana, 2GV6, prediluted, 790-4341; CD4, Ventana, SP35, prediluted, 790-4423; CD8, Ventana, SP57, prediluted, 790-4460; CD14, Abcam, 1H5D8, 200, ab181470; CD20, DAKO, L26, 200, M0755; Recombinant CD103, Abcam, SP301, 100, Ab227697; Cleaved Caspase-3, Cell Signaling Technology, Asp175, 100, 9661; CEA, Abcam, EPR20721, 100, ab207718; EpCAM, Ventana, Ber-EP4, prediluted, 760-4383; E-Cadherin, Ventana, 36, prediluted, 760-4497; Pan-Cytokeratin, Biorybt, C-11, 100, Orb43707; Granzyme B, Abcam, 100, Ab4059; ZO-1, Invitrogen, Z11A12, 50, 33-9100

2. Flow-cytometry antibodies: Antigen, Vendor, Clone, Fluorophore, Dilution, Reference;
IIFNγ, BD Biosciences, B27, BUV395, 200, 563563; CD69, BD Biosciences, FN50, BUV737, 200, 612817; CD8, BD Biosciences, SK1, BUV805, 200, 612889; Ki-67, Biolegend, Ki-67, BV421, 200, 350506; CD103, Biolegend, Ber-ACT8, BV421, 200, 350214; CD45, Biolegend, 2D1, BV510, 200, 368526; CD19, Biolegend, HIB19, BV605, 200, 302244; HLA-DR, Biolegend, L243, BV650, 200, 307650; CD11b, Biolegend, ICRF44, BV711, 200, 301344; CD45RA, Biolegend, HI100, BV786, 200, 304140; CD45RO, Biolegend, UCHL1, FITC, 200, 304204; TNFα, Biolegend, MAb11, PE, 200, 502909; Granzyme B, Biolegend, QA16A02, PE-Dazzle-594, 200, 372215; 41BB, Biolegend, 4B4-1, PE-Cy7, 200, 309818; IL-2, Biolegend, MQ1-17H12, APC, 200, 500310; CD3, Biolegend, HIT3a, Alexa Fluor 700, 200, 300324; Efluor780 Fixable L/D, Thermo, N/A, APC-H7, N/A, 65-0865-14

Validation

Secondary only and isotypes controls were applied during establishments of antibody staining. All primary antibodies were tested on human native tissue as validation for specific staining, and evaluated by a pathologist (Nadine Stokar) before applying them on organoids.

# Eukaryotic cell lines

Policy information about cell lines and Sex and Gender in Research

Cell line source(s)

Organoid cell lines were derived from primary surgical resections. Healthy organoids were derived from the healthy margin of tumour resections, whereas tumour organoids were derived from the malignant tissue. Information on the sex of the patients is available for each organoid line (Supplementary Table 1).

Authentication

Histological assessment and IHC staining for intestinal markers were performed by a trained pathologist to verify the intestinal epithelial origin of the organoids, and to verify the classification into healthy vs. transformed epithelium.

Mycoplasma contamination

All cell lines used were verified to be negative for mycoplasma before experimentation.

Commonly misidentified lines
(See ICLAC register)

No commonly misidentified cell lines were used.

# Flow Cytometry

## Plots

Confirm that:

☒ The axis labels state the marker and fluorochrome used (e.g. CD4-FITC).

☒ The axis scales are clearly visible. Include numbers along axes only for bottom left plot of group (a 'group' is an analysis of identical markers).

☒ All plots are contour plots with outliers or pseudocolor plots.

☒ A numerical value for number of cells or percentage (with statistics) is provided.

## Methodology

Sample preparation

PBMCs-organoid cocultures were digested into a single-cell suspension with accutase and filtered, subjected to flow cytometry staining (as detailed in Methods) and then acquired immediately

| | |
|---|---|
| Instrument | BD LSRFortessa X20 |
| Software | FowJo v10.8.1 |
| Cell population abundance | No cell populations were sorted. |
| Gating strategy | Provided in Supplementary Fig. 2. |

☒ Tick this box to confirm that a figure exemplifying the gating strategy is provided in the Supplementary Information.
